# The Release and Transformation of Sb and As Induced by Microorganisms in Antimony Smelting Soil

**DOI:** 10.3390/toxics14090759

**Published:** 2026-08-26

**Authors:** Yan Zheng, Zhihao Wu, Junhuan Wang, Hong Hou

**Affiliations:** 1 State Key Laboratory of Pollution Control and Resources Reuse, College of Environmental Science and Engineering, Tongji University, Shanghai 200092, China; zhengyan_@tongji.edu.cn; 2 State Key Laboratory of Environmental Criteria and Risk Assessment, Chinese Research Academy of Environmental Sciences, Beijing 100012, China; wuzh7714@163.com; 3 College of Water Sciences, Beijing Normal University, Beijing 100875, China; 4 School of Ocean Food and Biological Engineering, Jiangsu Ocean University, Lianyungang 222005, China

**Keywords:** antimony smelting soil, antimony, arsenic, microbial oxidation, biogeochemical cycling

## Abstract

Mining and smelting activities have accelerated the release of antimony (Sb) and arsenic (As) into the environment, resulting in high ecological risks. Microorganisms are considered key drivers of Sb and As cycling. However, their effects on Sb/As transformation in antimony smelting soils remain poorly understood. In this study, an indigenous microbial consortium capable of oxidizing both Sb and As was obtained from antimony smelting soil and used to investigate the interactions between microorganisms and Sb-As co-contaminated soils. The results showed that the functional microbial consortium enhanced Sb and As release from the soil by increasing the system pH from 7.41 to 8.89, thereby promoting the dissolution of solid-phase Sb and As. The concentrations of total dissolved Sb and As reached 246.47 and 30.5 µmol L^−1^ by day 16, respectively, and the released Sb(III) and As(III) were completely oxidized to Sb(V) and As(V). In the abiotic control, the corresponding concentrations of total dissolved Sb and As were 95.89 and 12.08 µmol L^−1^, respectively, with an As(III) concentration of 3.54 µmol L^−1^. Sequential extraction results revealed that microbial activity altered the speciation distribution of Sb and As in soils, decreasing the proportions of easily exchangeable and specifically surface-bound fractions while increasing the residual fractions. X-ray photoelectron spectroscopy (XPS) analysis further showed that, compared with the original soil, the functional microbial consortium increased the proportions of Sb(V) and As(V) in the solid phase from 34.79% to 40.54% and from 9.53% to 15.96%, respectively, suggesting that microbial activity might promote the precipitation of Sb(V) and As(V), thereby facilitating the re-immobilization of a fraction of the released Sb and As. Furthermore, the succession of microbial communities during the interaction process was investigated. The release of Sb and As from soil increased the abundance of microorganisms related to Sb/As metabolism. Spearman correlation analysis suggested that *Arenimonas*, *Luteimonas*, *Arthrobacter*, and *Brevundimonas* were likely involved in Sb(III)/As(III) oxidation, whereas *Devosia* and *Aminobacter* contributed to the elevation of system pH. This study provides insights into the microbial-mediated transformation, migration, and oxidation of Sb and As in soils.

## 1. Introduction

Antimony (Sb) and arsenic (As) are metalloids widely present in the natural environment. The U.S. Environmental Protection Agency has designated them as a priority pollutant and a suspected carcinogen, respectively [1,2]. As Group 15 elements in the periodic table, Sb and As exhibit similar geochemical behaviors and frequently co-occur in sulfide minerals, resulting in widespread co-contamination of Sb and As in mining areas [3]. As a major producer and consumer of metal(loid)s worldwide, China possesses abundant Sb resources, ranking first globally in Sb reserves, production, and consumption. Currently, exploited antimony deposits in China are mainly distributed in the southwestern region, which accounts for 86.1% of the national Sb reserves [4,5,6]. Previous investigations at antimony mining and smelting sites in southwestern China demonstrate that soil Sb and As concentrations can reach hundreds to tens of thousands of mg kg^−1^, greatly exceeding regional soil background values [6,7,8]. Therefore, elucidating the geochemical behaviors of Sb and As in these contaminated environments is essential for accurate environmental risk assessment and the development of effective remediation strategies. However, the migration and transformation mechanisms of Sb and As in such environments remain poorly understood.

Microorganisms play fundamental roles in regulating soil biogeochemical cycles, and indigenous microbial communities inhabiting heavy metal(loid)-contaminated smelting sites can substantially influence the geochemical transformation of these pollutants, providing potential opportunities for bioremediation. Previous studies have demonstrated that microorganisms mediate key Sb/As biotransformation processes, including oxidation, reduction, and methylation [9,10], thereby regulating the toxicity, mobility, and bioavailability of Sb and As [11,12]. The environmental toxicity of Sb and As is largely dependent on their redox states, with the reduced species Sb(III) and As(III) generally exhibiting greater toxicity than the oxidized species Sb(V) and As(V) [13,14,15]. Accordingly, microbial oxidation of Sb/As is considered an important detoxification process during their biogeochemical cycling. To date, numerous Sb(III)- and As(III)-oxidizing bacteria have been isolated from various environments, including soils and mine sediments [16,17,18], most of which belong to the phylum *Proteobacteria* [16]. Although microbial oxidation of dissolved Sb(III)/As(III) has been extensively investigated under laboratory conditions [19,20], the mechanisms by which microorganisms regulate Sb/As migration and transformation in soil environments remain insufficiently understood.

In this study, an indigenous microbial consortium capable of oxidizing both Sb(III) and As(III) was used to investigate its interactions with antimony smelting soil using a microcosm approach. The variations in dissolved Sb and As concentrations was determined through aqueous-phase analysis, while changes in Sb and As speciation and mineralogical characteristics in the solid phase were analyzed using multiple analytical techniques. Furthermore, microbial community succession during incubation was characterized using 16S rRNA gene sequencing. This study aims to elucidate the mechanisms by which microorganisms regulate the migration and transformation of Sb and As in antimony smelting soils.

## 2. Materials and Methods

### 2.1. Sampling Site

**Figure 1:**
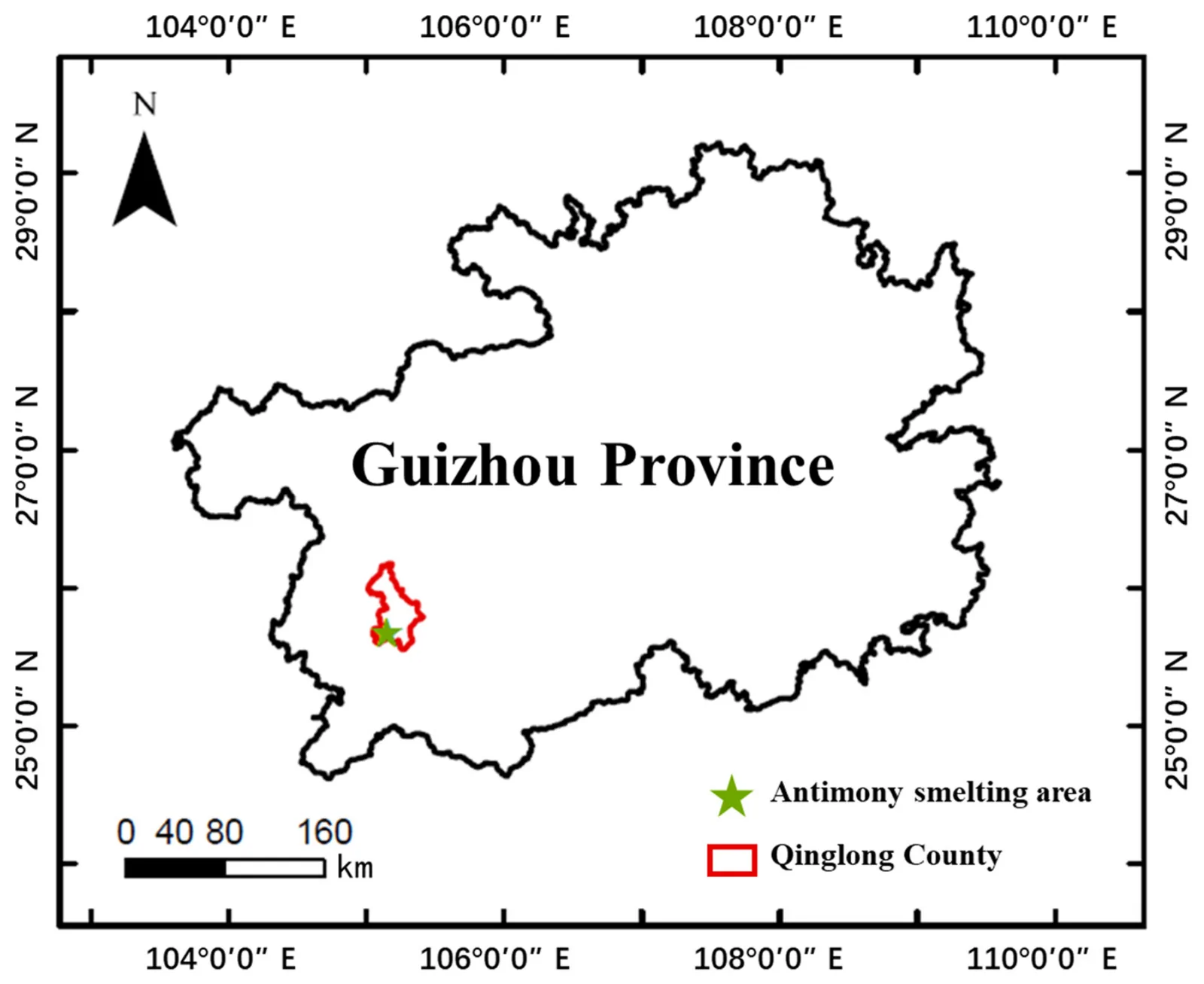
Location of the abandoned antimony smelting site.

The Qinglong Antimony Mine contains approximately 199,600 tons of Sb ore reserves, ranking as the third-largest antimony deposit in China [4,21]. Located in Qinglong, Guizhou Province, China, the site experiences a subtropical monsoon climate, with an annual mean temperature of 14.1 °C and an average annual precipitation of 1460 mm. Surface soil samples (0–2 cm depth) were collected from an abandoned antimony smelting site in the Qinglong antimony mining area (Figure 1) using a sterile stainless-steel spoon. All soil samples were placed in sterile bags, immediately frozen on dry ice, transported to the laboratory under cold conditions, and stored at −80 °C until further treatment and analysis. The physicochemical properties of the soil sample are shown in Table 1.

### 2.2. Experimental Setup

The microbial consortium used in this study was obtained from soil collected at the Qinglong smelting area through enrichment cultivation. It was capable of completely oxidizing 1000 μM Sb(III) and 1000 μM As(III) to Sb(V) and As(V) within 72 and 24 h, respectively. The refrigerated microbial consortium was thawed at room temperature for 30 min and subsequently cultivated in 100 mL of sterile chemically defined medium (CDM) [22] in shaking flasks at 30 °C and 150 rpm in the dark (the detailed composition of CDM is presented in Table S1). The initial pH of the CDM was adjusted to 7.0. After a 24 h preculture, the bacterial inoculum was collected and used for subsequent microcosm experiments.

For the soil microcosm incubation, 30 g of soil was ground to pass through a 200-mesh sieve, added to 300 mL of CDM, and sterilized at 121 °C for 30 min. The initial pH of the CDM was adjusted to 7.0. After cooling, the bacterial culture was inoculated at a volume ratio of 1% (the initial cell density of the bacterial inoculum was approximately 1.98 × 10^7^ CFU mL^−1^). A sterile group without microbial inoculation was established as the abiotic control. Both the inoculated treatment and the abiotic control were incubated at 30 °C and 150 rpm in the dark under aerobic conditions. Samples were collected every two days for solution chemical analysis and every four days for microbial community analysis. All experiments were conducted in triplicate. Sterile deionized water was periodically added to compensate for evaporative and sampling losses. (Detailed initial experimental conditions are presented in Table S2).

### 2.3. Chemical Analysis

The pH of the solution was measured using a calibrated pH meter (Mettler-Toledo, Columbus, OH, USA). The pH meter was calibrated before each measurement series using three standard buffer solutions (pH 4.01, 7.00, and 9.21, Mettler-Toledo) covering the expected pH range of the samples. The dissolved Sb and As redox species (Sb(III), As(III), Sb(V) and As(V)) were analyzed using liquid chromatography–atomic fluorescence spectrometry (LC-AFS) (Beijing Jitian, Beijing, China). The redox species of Sb and As in the solid phase were extracted using citric acid according to the procedure described by Fuentes et al. [23], and subsequently analyzed by LC-AFS. The total concentrations of Sb and As in soil were determined following HCl-HF-H_2_O_2_-HNO_3_ digestion and subsequent analysis by inductively coupled plasma optical emission spectroscopy (ICP-OES) (Agilent, Boulder, CO, USA). A modified five-stage sequential extraction procedure, based on Wenzel et al. [24], was employed to determine the distribution of different Sb and As fractions in soil samples (detailed information is presented in Table S3), followed by analysis using ICP-OES.

### 2.4. Solid-Phase Characterization

To further elucidate the transformation of Sb and As speciation, the original soil sample and solid residues collected from the microcosm experiments were characterized by X-ray photoelectron spectroscopy (XPS) (Malvern Panalytical, Almelo, Netherlands). XPS is a semi-quantitative technique. In this study, it was used to determine the relative proportions of Sb(III)/Sb(V) and As(III)/As(V) based on peak-area deconvolution. The morphology and elemental composition of the original soil sample and solid residues were examined using scanning electron microscopy (SEM) (Zeiss, Oberkochen, Germany) coupled with energy-dispersive spectrometry (EDS) (Oxford Instruments, Oxford, UK).

### 2.5. Microbiological Analysis

The microbial community was analyzed based on 16S rRNA gene amplicon sequencing of bacteria. The raw sequencing reads were quality-filtered using fastp (version 0.20.0) [25], and bases with quality scores below 20 at the read ends were removed. The resulting high-quality reads were subsequently merged using FLASH (version 1.2.7) [26]. Operational taxonomic units (OTUs) were clustered from the processed sequences using UPARSE (version 7.1) [27] at a 97% sequence similarity threshold. Taxonomic annotation of OTUs was conducted using RDP Classifier (version 2.11) [28] based on the SILVA 16S rRNA gene database (release v138). Microbial alpha diversity analysis and Spearman correlation analysis were conducted on the Majorbio Cloud Platform (https://www.majorbio.com (accessed on 18 June 2026)).

## 3. Results and Discussion

### 3.1. The Variations in pH in Microcosms

**Figure 2:**
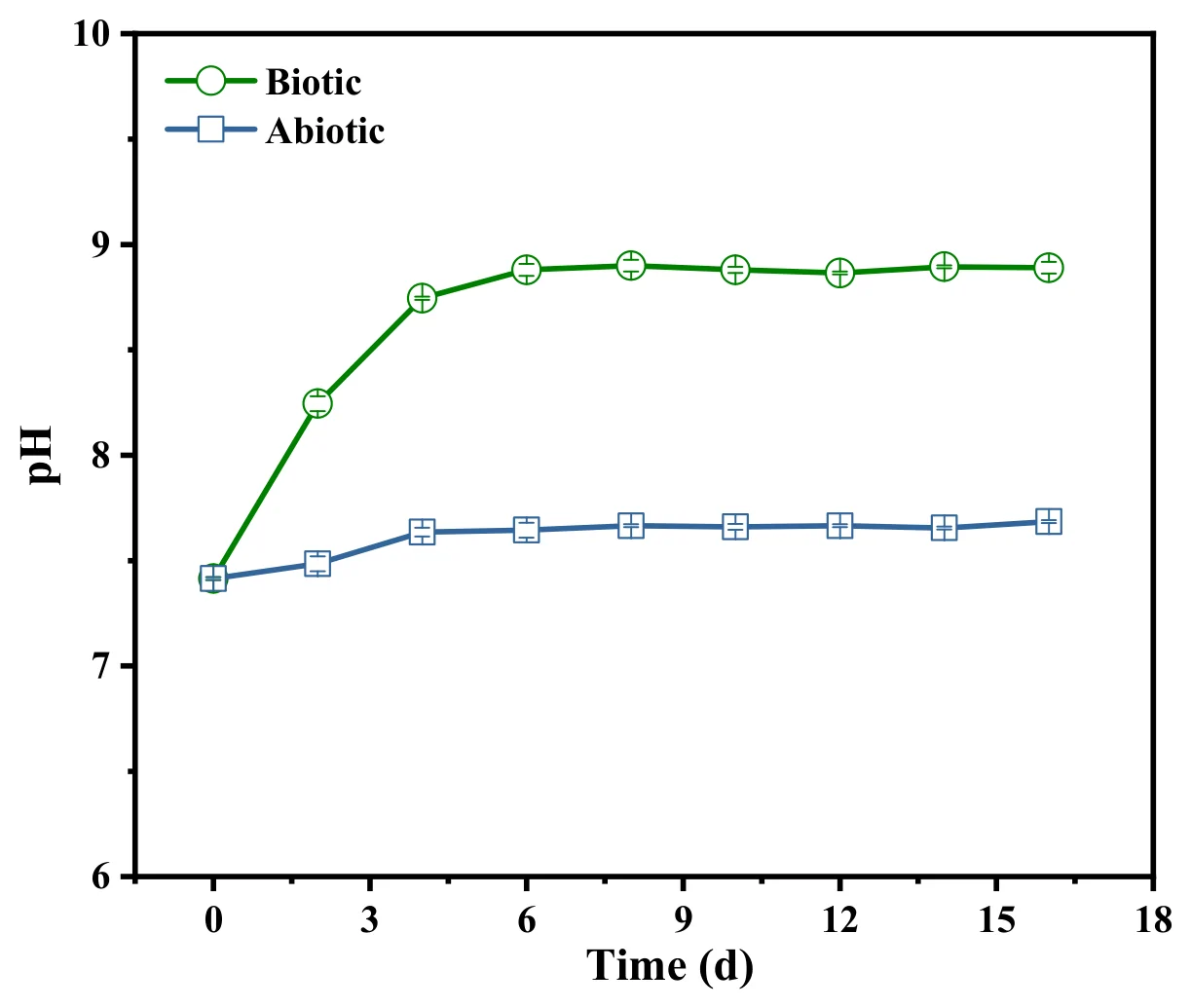
Variations in pH during microcosm incubation. Data are presented as mean ± standard deviation (*n* = 3).

The temporal variations in solution pH for the inoculated treatment and abiotic control are presented in Figure 2. Microbial inoculation induced a rapid increase in system pH within 6 days, reaching a maximum value of 8.89, whereas the abiotic control exhibited only a slight initial increase and subsequently stabilized at 7.64. As shown in Table 1, the pH of the antimony smelting soil used for the microcosm experiments was 7.39, further confirming that the increase in pH in the microbial treatment was regulated by microbial activity rather than by the intrinsic soil properties. Soil pH is a key factor controlling the solubility and mobility of Sb and As, as acidic conditions promote the protonation of metal(loid) oxide surfaces, generating positive charges that enhance the electrostatic adsorption of Sb and As [29,30]. An increase in soil pH can reduce the adsorption capacity of soils for Sb and As, thereby facilitating their dissolution from mineral phases [30,31,32]. Therefore, the elevated pH induced by microbial inoculation likely contributed to the enhanced release of Sb and As from the soil.

### 3.2. Behavior of Aqueous-Phase Sb and As

**Figure 3:**
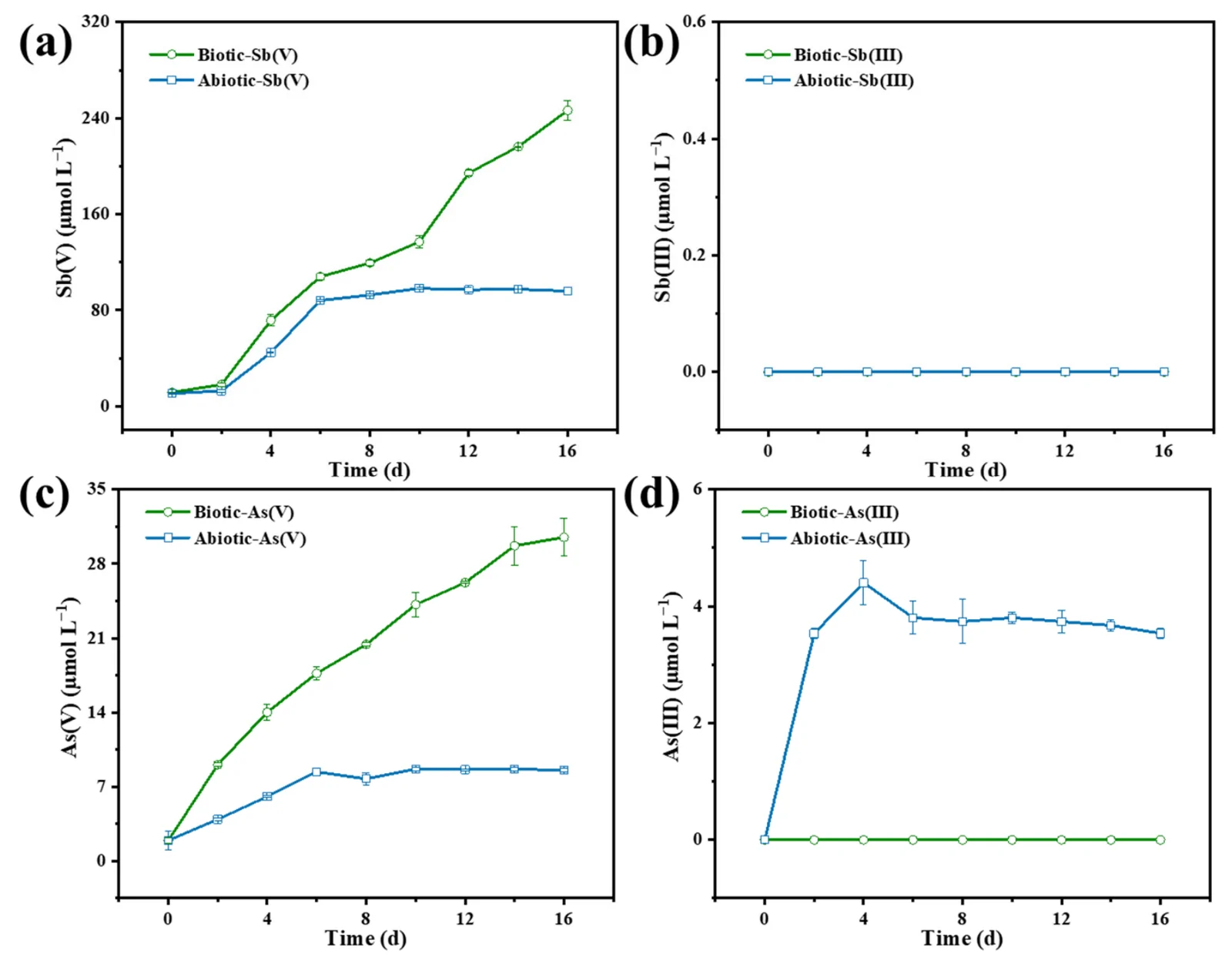
Variations in dissolved (**a**) Sb(V), (**b**) Sb(III), (**c**) As(V), and (**d**) As(III) concentrations during microcosm incubation. Data are presented as mean ± standard deviation (*n* = 3).

As shown in Figure 3a,b, dissolved Sb(III) remained below the detection limit in both the inoculated and abiotic systems, whereas dissolved Sb(V) concentrations continuously increased throughout the incubation period. By day 16, the final Sb(V) concentrations reached 95.89 and 246.47 µmol L^−1^ in the abiotic control and microbial inoculation systems, respectively, representing a 1.57-fold higher release in the inoculated treatment. Similarly, dissolved As(V) concentrations increased progressively in all treatments (Figure 3c), reaching final concentrations of 8.54 and 30.5 µmol L^−1^ in the abiotic and inoculated systems, respectively, corresponding to a 2.57-fold increase. Notably, As(III) was not detected in the microbial treatment, whereas a slight accumulation of As(III) was observed in the abiotic control during the early incubation stage, with a maximum concentration of 3.54 µmol L^−1^ (Figure 3d). Previous characterization of soils from the Qinglong smelting site demonstrated that Sb and As predominantly occur in pentavalent forms, with relatively lower proportions of trivalent species [7,8]. Considering the results from the abiotic control, the absence of detectable Sb(III)/As(III) in the microbial treatment suggests that the dissolved Sb(III)/As(III) released from the soil was rapidly oxidized to Sb(V)/As(V) by microorganisms. In addition, dissolved Sb concentrations consistently exceeded those of As regardless of microbial inoculation. This observation is consistent with the findings of Li et al. [33], who investigated Sb and As transport in Qinglong smelting soils using a two-dimensional tank under wet–dry cycling conditions and reported higher mobility of Sb than As, likely due to differences in their total concentrations. These results demonstrate that microbial inoculation enhanced the mobilization of Sb and As from contaminated soil while facilitating the complete oxidation of released Sb(III) and As(III) to their oxidative forms, with Sb exhibiting greater mobility than As. Notably, based on the pH variations shown in Figure 2, this microbially enhanced release of Sb and As from the soil was likely driven by the increase in system pH.

### 3.3. Changes in Solid-Phase Sb and As Species

**Figure 4:**
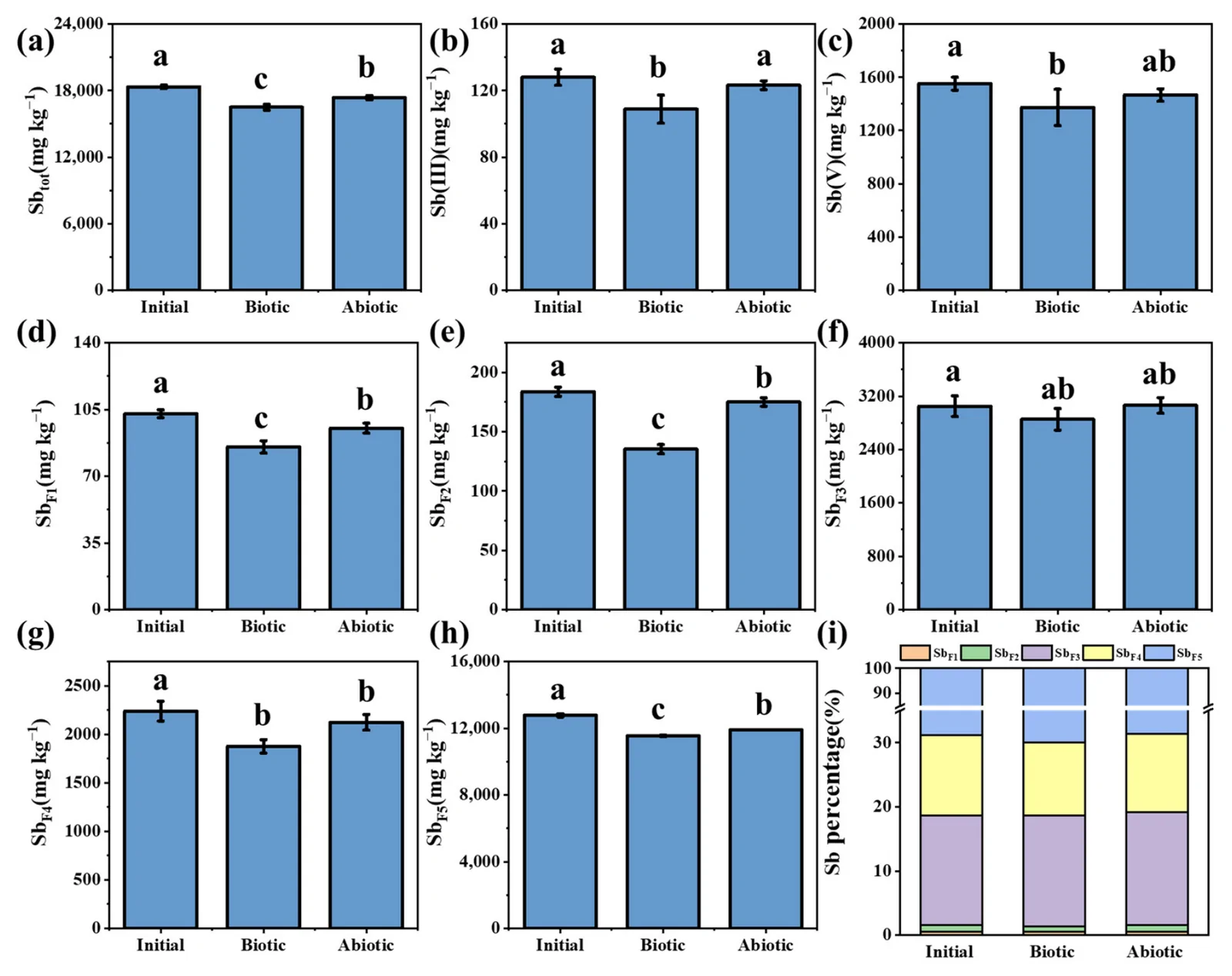
(**a**) Total Sb, (**b**) Sb(III) from citric acid extraction, (**c**) Sb(V) from citric acid extraction, (**d**) easily exchangeable Sb, (**e**) specifically surface-bound Sb, (**f**) amorphous hydrous oxide-bound Sb, (**g**) crystalline hydrous oxide-bound Sb, (**h**) residual Sb, and (**i**) Sb composition in the initial soil and soil samples collected in different treatments. (The letters a, b, c indicate significant differences between groups, *p* < 0.05).

**Figure 5:**
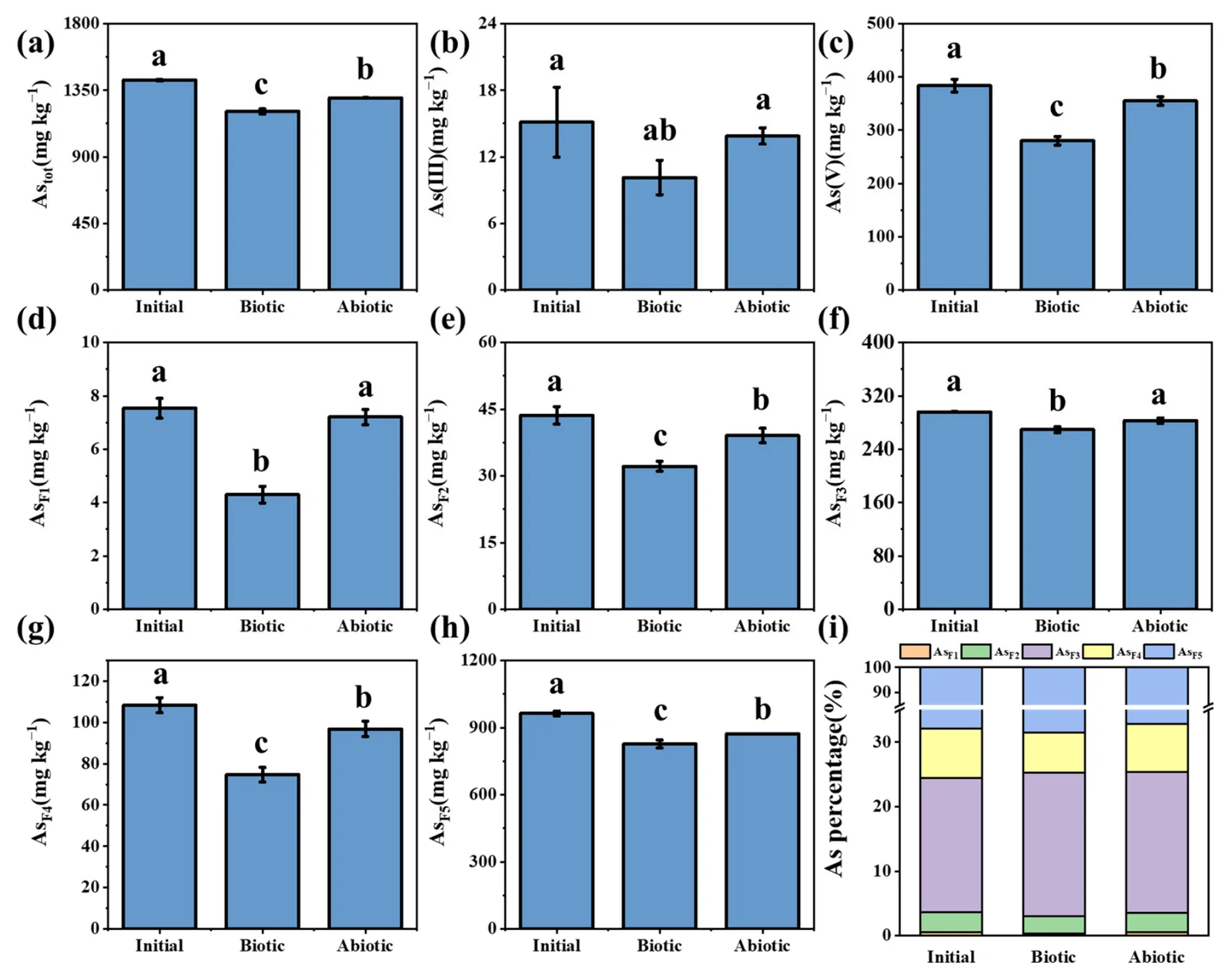
(**a**) Total As, (**b**) As(III) from citric acid extraction, (**c**) As(V) from citric acid extraction, (**d**) easily exchangeable As, (**e**) specifically surface-bound As, (**f**) amorphous hydrous oxide-bound As, (**g**) crystalline hydrous oxide-bound As, (**h**) residual As, and (**i**) As composition in the initial soil and soil samples collected in different treatments. (The letters a, b, c indicate significant differences between groups, *p* < 0.05).

The solid phase of soil serves as both the primary source of dissolved Sb and As and an important sink for their immobilization. Therefore, the variations in solid-phase Sb and As speciation before and after microbial treatment were investigated in this study. As shown in Figure 4 and Figure 5, microbial inoculation significantly decreased the total concentrations of Sb and As in soil (Figure 4a and Figure 5a), which was consistent with the increased dissolved Sb and As concentrations observed in the microbial systems, further demonstrating that microorganisms promoted the release of Sb and As from the solid phase into the aqueous phase. The five-step sequential extraction results showed that, after microbial inoculation, the contents of easily exchangeable Sb, specifically surface-bound Sb, and residual Sb, as well as the contents of easily exchangeable As, specifically surface-bound As, amorphous hydrous oxide-bound As, and crystalline hydrous oxide-bound As, were significantly lower than those in the initial soil and abiotic control soils (Figure 4d,e,h and Figure 5d–g). These results demonstrate that microbial activity promoted the release of multiple Sb and As fractions. However, the dominant sources contributing to Sb and As mobilization were not identical. The release of As primarily originated from non-residual fractions possessing relatively higher mobility than the residual fraction, which is consistent with the general rule that more mobile metal(loid) fractions are preferentially released compared with the relatively stable residual fraction [34]. In contrast, the mobilization of Sb was derived not only from bioavailable Sb fractions but also substantially from the residual Sb.

The antimony smelting site where the soil samples were collected was historically involved in Sb production through pyrometallurgical processing using stibnite as the primary ore source. Therefore, the residual Sb fraction in these soils may contain abundant Sb-bearing minerals. Previous studies have demonstrated that microorganisms can promote the dissolution and oxidation of Sb in stibnite [32,35,36,37]. Accordingly, the significant decrease in residual Sb following microbial treatment was likely attributable to the microbial-mediated dissolution of Sb-bearing minerals in the soil. The analysis of Sb and As redox species extracted by citric acid further revealed that the contents of Sb(III) and As(V) in microbial-treated soil were significantly lower than those in the initial soils and abiotic control (Figure 4b and Figure 5c), indicating that microorganisms preferentially promoted the release of solid-phase Sb(III) and As(V) compared with Sb(V) and As(III). This observation is consistent with previous studies [38,39,40], suggesting that Sb(V) exhibits greater mobility than Sb(III), whereas As(III) is more mobile than As(V) in soil environments. Overall, microbial activity altered the distribution characteristics of Sb and As in the soil, resulting in decreased proportions of bioavailable (easily exchangeable and specifically surface-bound) fractions accompanied by an increased proportion of residual Sb/As (Figure 4i and Figure 5i).

**Figure 6:**
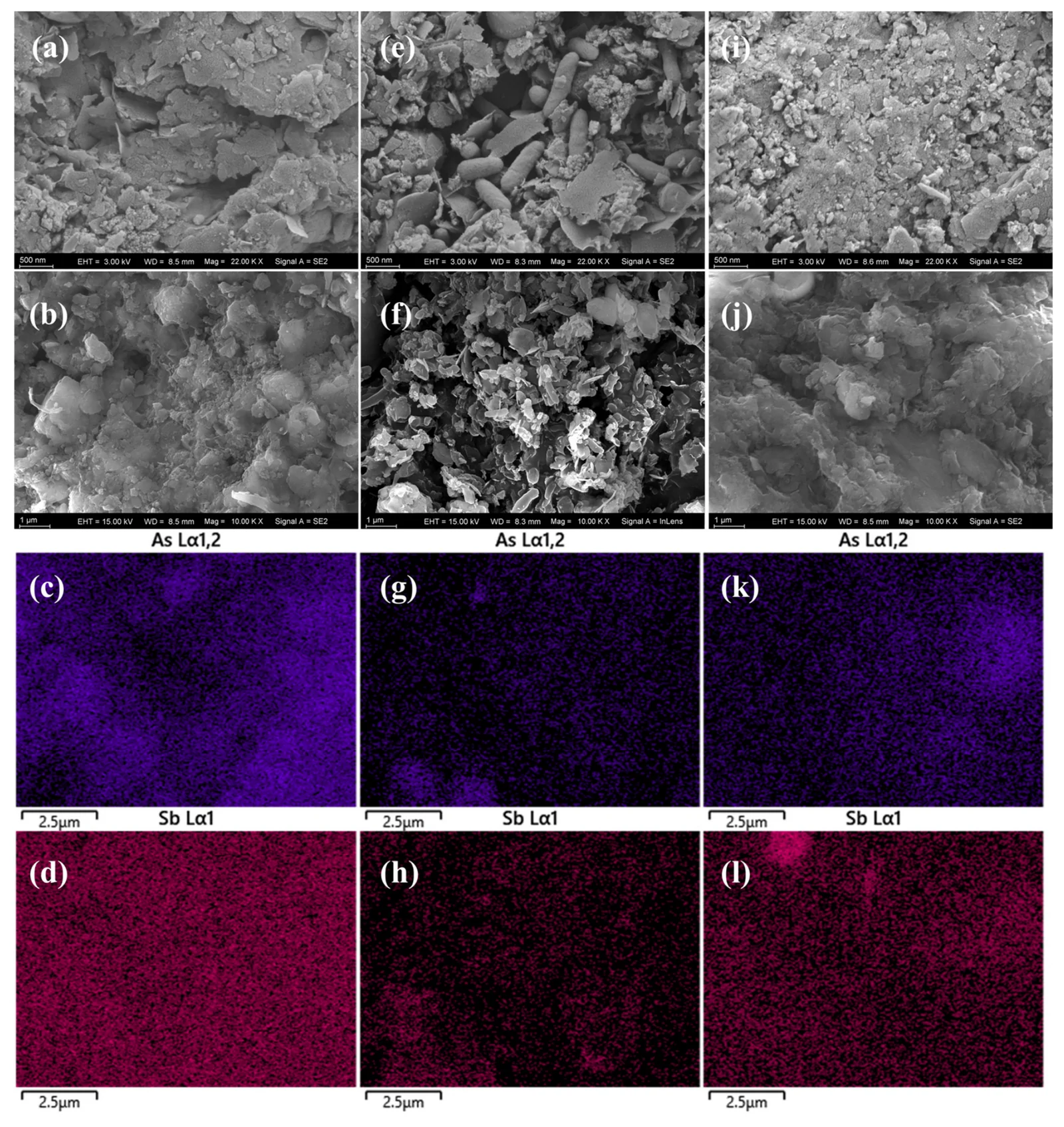
SEM and SEM-EDS mapping images of (**a**,**b**) initial soil, (**e**,**f**) solid sample collected from the biotic system, and (**i**,**j**) solid sample collected from the abiotic system. The distribution of Sb and As in (**c**,**d**) initial soil, (**g**,**h**) solid sample collected from the biotic system, and (**k**,**l**) solid sample collected from the abiotic system.

**Figure 7:**
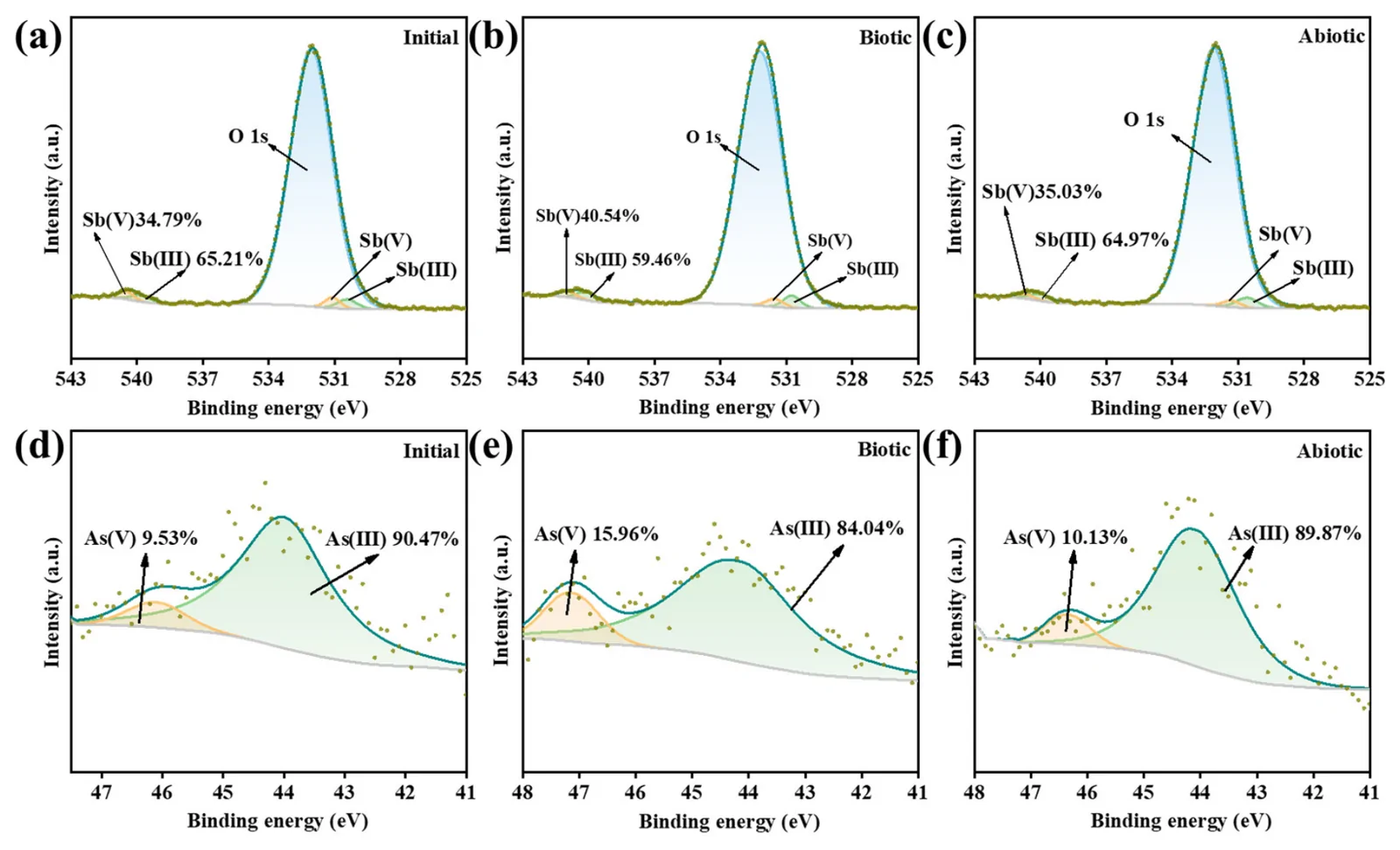
XPS spectrum of (**a**–**c**) Sb and (**d**–**f**) As in the initial soil and soil samples collected in different treatments.

As shown in Figure 6, SEM images revealed that microbial inoculation increased the porosity of the soil compared with the initial soil and the soil in abiotic control, with numerous rod-shaped microorganisms observed within the pore spaces. EDS elemental mapping further demonstrated a reduced distribution of Sb and As in the inoculated soil. This observation was consistent with the variations in Sb and As concentrations in both aqueous and solid phases (Figure 3, Figure 4 and Figure 5), confirming that microorganisms promoted the dissolution and release of Sb and As from the soil. XPS characterization indicated that microbial activity altered the redox speciation of Sb and As in the solid phase, resulting in increased proportions of Sb(V) and As(V) compared with those in the initial soil and the soil in abiotic control (Figure 7). The contents of Sb and As redox species extracted by citric acid demonstrated that microorganisms preferentially facilitated the release of Sb(III) and As(V) rather than Sb(V) and As(III) from the soil (Figure 4b and Figure 5c). For As, this observation appeared inconsistent with the XPS results, as enhanced release of As(V) would theoretically decrease its relative proportion in the solid phase. This discrepancy may be attributed to the microbial-mediated re-precipitation and immobilization of released As(V). In contrast, the distribution of Sb(V) determined by citric acid extraction was consistent with the XPS results, indicating that the microbially promoted release of Sb(III) was a major contributor to the increased proportion of Sb(V) in the solid phase. According to the aqueous-phase chemical results, the released Sb(III) was rapidly and completely oxidized to Sb(V) by microorganisms. Previous studies have demonstrated that microorganisms can mediate the formation of Sb(V)-bearing secondary mineral precipitates [32]. Therefore, the possibility that microbially induced Sb(V) precipitation contributed to the increased proportion of solid-phase Sb(V) cannot be excluded. Moreover, microbial activity increased the proportions of residual Sb and As in the soil (Figure 4i and Figure 5i).

Combined with the solution chemistry results and the sequential extraction of solid-phase Sb and As after experiments, the increase in the residual fractions can likely be attributed, on the one hand, to the microbially promoted release of the more labile easily exchangeable and specifically surface-bound Sb and As fractions and, on the other hand, to the formation of Sb(V)/As(V) precipitates. Loni et al. reported that the Sb-oxidizing bacterium *Paracoccus versutus* XT0.6 mediated the dissolution and oxidation of Sb from stibnite, followed by the formation of the secondary Sb(V)-bearing mineral NaSb(OH)_6_ from a fraction of the dissolved Sb(V) [32]. In addition, Sb(V) in soils has been demonstrated to readily interact with alkali metal ions such as Ca, Mg, Na, and K, forming alkali metal hydroxyantimonate salts with exceptionally low solubilities under ambient conditions, such as mopungite [NaSb(OH)_6_] and potassium hexahydroxoantimonate [KSb(OH)_6_] [29]. Similarly, As(V) can readily react with Ca and Fe in environmental matrices to form relatively stable precipitates, such as Ca_3_(AsO_4_)_2_ and FeAsO_4_·2H_2_O [41]. Previous studies have further demonstrated that, under high-pH conditions and high Ca/As molar ratios, As(V) can be stabilized predominantly in the form of calcium arsenate minerals [42]. As shown in Table 1, the soil used in this study contained abundant K, Ca, Na, and Fe, while microbial activity promoted the oxidation of Sb(III) and As(III) and increased the system pH (Figure 2), collectively creating favorable conditions for Sb/As precipitation. Based on these findings, a possible mechanism underlying the microbially induced increase in residual Sb and As can be proposed. Microorganisms first promoted Sb/As release from the soil by increasing the system pH and subsequently completely oxidized the released Sb(III) and As(III) to Sb(V) and As(V). The resulting Sb(V) and As(V) then interacted with K, Ca, Na, and Fe in the soil to form precipitates, thereby promoting their re-immobilization. Consequently, the release of the more labile easily exchangeable and specifically surface-bound Sb/As fractions, together with the precipitation of dissolved Sb(V) and As(V), jointly increased the relative proportions of residual Sb and As and thereby altered their distribution characteristics in the soil. However, these results have limitations regarding the identification of Sb/As mineral phases. Future studies should employ more advanced and precise techniques to further verify the Sb(V)/As(V) precipitates formed under microbial mediation.

### 3.4. Microbial Response to Sb and As Release

**Figure 8:**
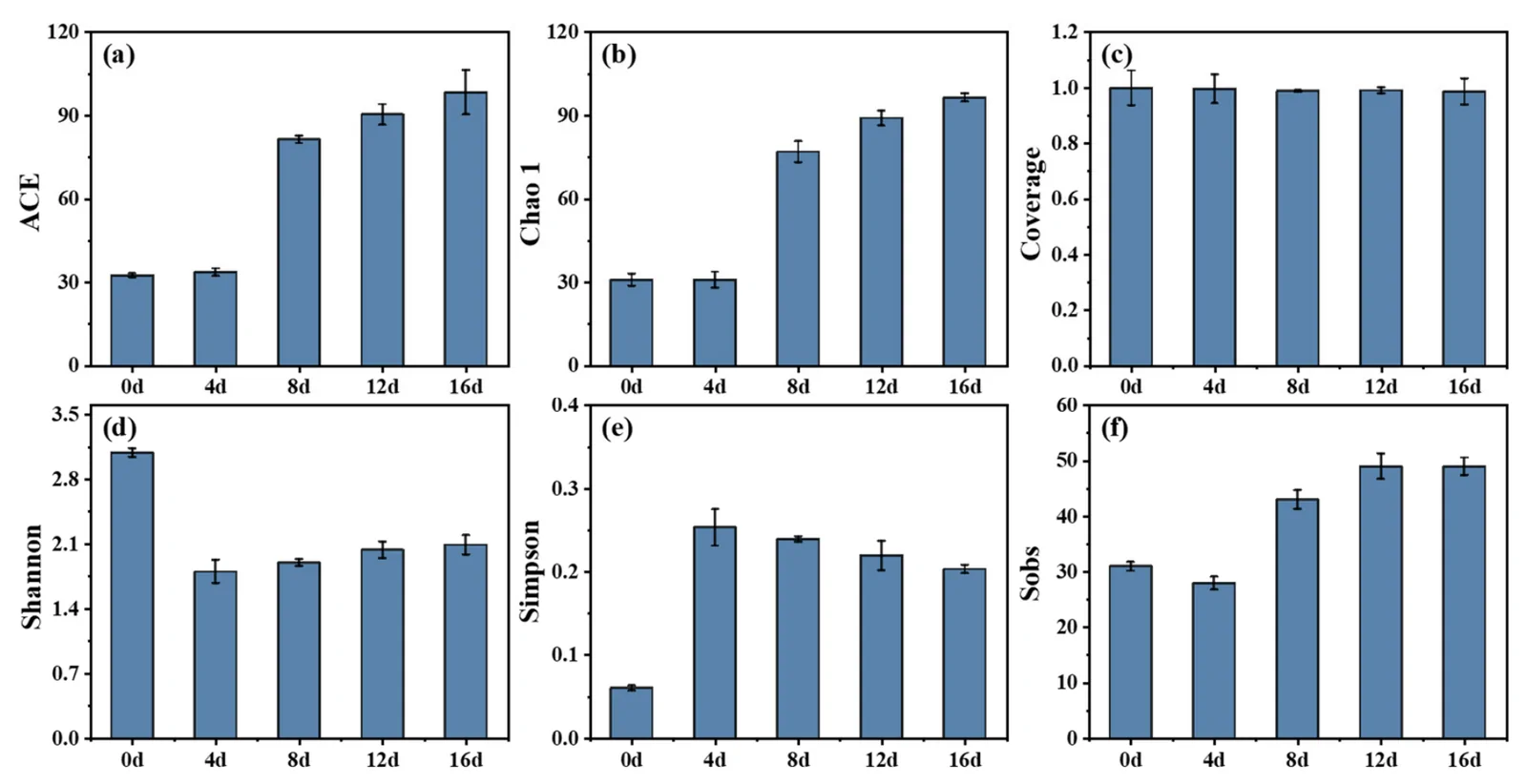
Alpha-diversity indices of the microbial community during the microorganism–soil interaction. (**a**) ACE, (**b**) Chao 1, (**c**) Coverage, (**d**) Shannon, (**e**) Simpson, and (**f**) Sobs.

The succession of the microbial community during its interaction with antimony smelting soil was investigated. As shown in Figure 8, alpha-diversity analysis of the microbial community in the inoculated microcosms revealed that, compared with the initial stage, microbial community richness increased after 8 days of co-incubation with soil, as indicated by the ACE and Chao1 indices. However, microbial community diversity decreased after 4 days of incubation, based on the Shannon and Simpson indices. These results suggest that the presence of antimony smelting soil inhibited the growth of certain microorganisms while promoting the enrichment of others. Previous studies have reported that soils from the Qinglong antimony smelting area are contaminated not only with Sb and As but also with various other metal(loid)s [7]. Therefore, the reduction in microbial community diversity in the microcosm systems may be attributed to the combined stress imposed by Sb, As, and other metal(loid) contaminants present in the soil. It should be noted that the above findings were obtained from microcosms established using CDM. However, not all microorganisms in soil environments are culturable in CDM, and thus the use of this medium may also have contributed to the observed reduction in microbial community diversity.

**Figure 9:**
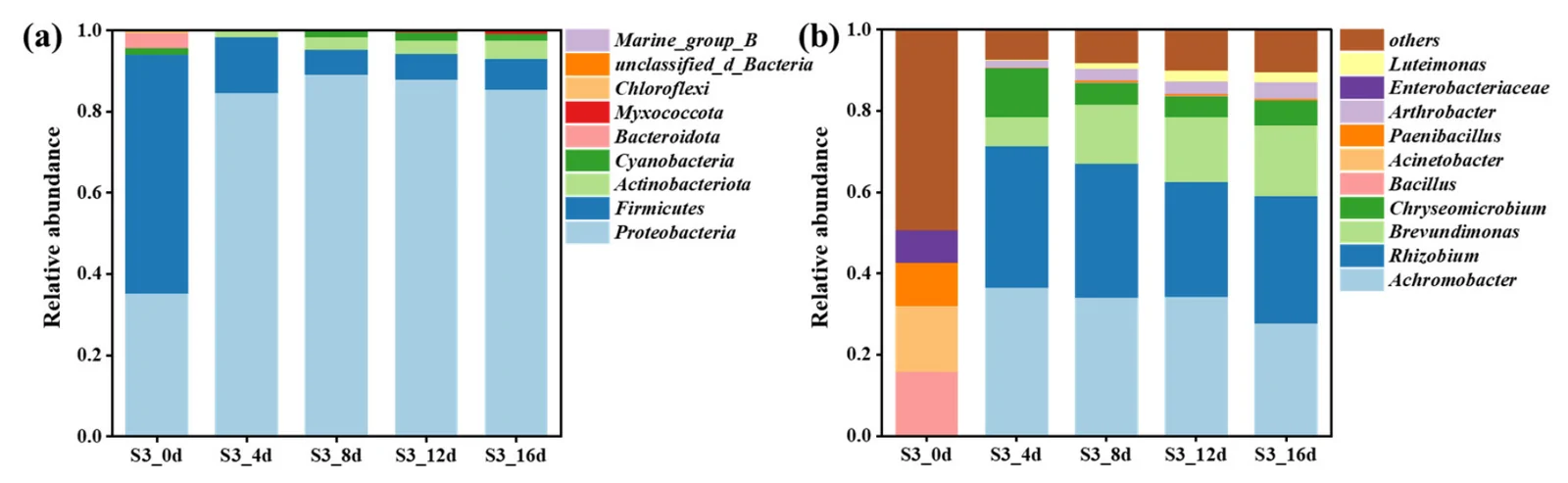
Microbial community structure during the microorganism–soil interaction. (**a**) Phylum level and (**b**) genus level.

**Figure 10:**
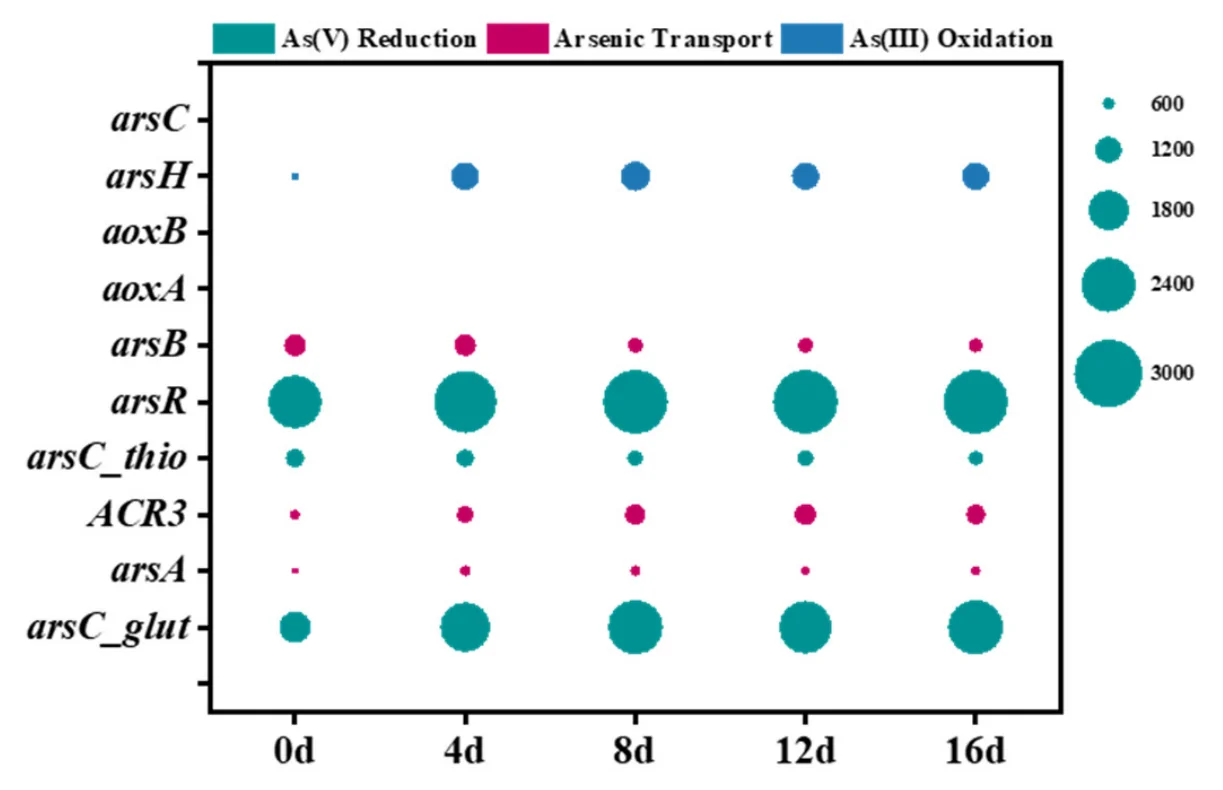
Potential Sb/As-related functional genes during the microorganism–soil interaction.

As shown in Figure 9, at the phylum level, the dominant microbial communities shifted from *Firmicutes* (58.69%), *Proteobacteria* (35.22%), and *Bacteroidota* (3.74%) at the initial stage to *Proteobacteria* (85.4%), *Firmicutes* (7.61%), and *Actinobacteriota* (4.5%) after incubation. The relative abundances of *Firmicutes*, *Bacteroidota*, and *Chloroflexi* gradually decreased, whereas those of *Proteobacteria*, *Actinobacteriota*, and *Myxococcota* progressively increased. At the genus level, the dominant genera gradually changed from *Acinetobacter* (16.19%), *Bacillus* (15.78%), *Paenibacillus* (10.59%), and *Enterobacteriaceae* (8.03%) at the initial stage to *Rhizobium* (31.49%), *Achromobacter* (27.61%), *Brevundimonas* (17.3%), and *Chryseomicrobium* (6.09%) after incubation. The relative abundances of *Acinetobacter*, *Bacillus*, and *Paenibacillus* gradually decreased, whereas those of *Rhizobium*, *Achromobacter*, and *Brevundimonas* progressively increased. These results indicate that the dissolution and release of Sb and As from antimony smelting soil drove microbial community succession, and the enriched dominant genera exhibited strong adaptability to Sb/As contamination. Deng et al. analyzed the phylogenetic relationships of 88 previously reported Sb-oxidizing bacteria and found that 87.5% belonged to the phylum *Proteobacteria* and 4.2% to *Actinobacteriota* [16]. In the present study, the relative abundance of *Proteobacteria* progressively increased with incubation time and ultimately became the dominant phylum. In addition, *Rhizobium*, the most abundant dominant genus, has been previously reported to possess abundant genes associated with Sb/As metabolism [43]. These findings indicate that the microbial taxa enriched at different taxonomic levels were closely associated with Sb/As metabolism, suggesting that antimony smelting soil selectively drove the enrichment of microorganisms involved in Sb/As transformation. As shown in Figure 10, PICRUSt prediction based on 16S rRNA sequencing revealed 10 potential functional genes associated with Sb/As metabolism in the microorganism–soil system. Among these genes, eight potential Sb/As metabolism-related genes, including Sb(III)/As(III) oxidation genes (*aoxA*, *aoxB*, and *arsH*) and Sb/As transport genes (*arsA* and *ACR3*), exhibited increased abundances after 4 days of incubation. This result was consistent with the changes in microbial community composition, further indicating that microorganisms enriched during incubation were highly associated with Sb/As metabolic processes.

### 3.5. Spearman Correlation Analysis

**Figure 11:**
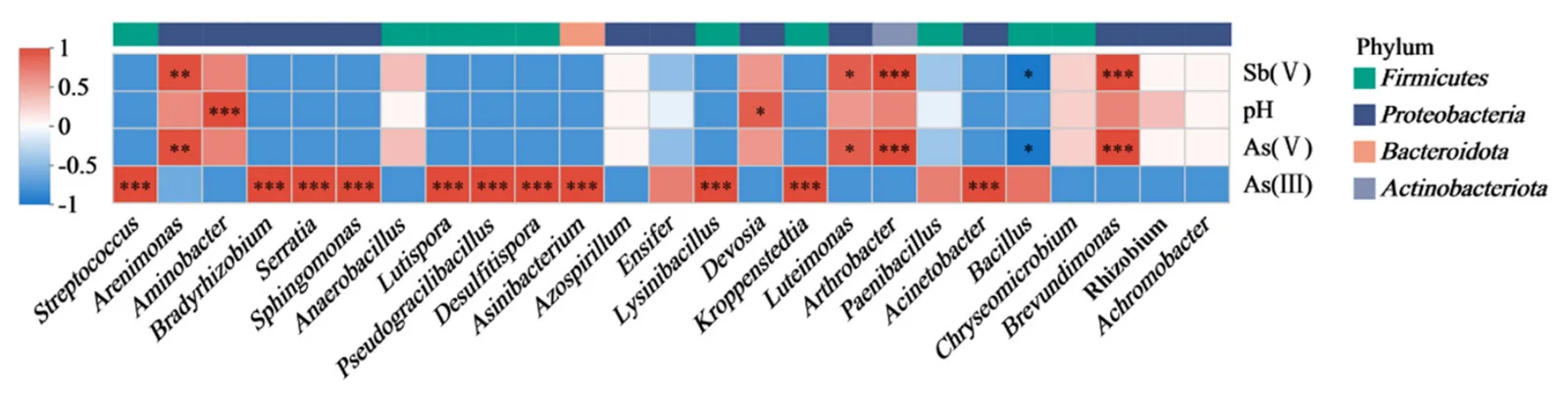
Relationships between chemical parameters and microorganisms. The *, **, and *** denote statistical significance at *p* < 0.05, <0.01, and 0.001, respectively.

As shown in Figure 11, Spearman correlation analysis revealed that *Arenimonas*, *Luteimonas*, *Arthrobacter*, and *Brevundimonas* were significantly and positively correlated with Sb(V) and As(V) concentrations in the system, whereas *Devosia* and *Aminobacter* exhibited significant positive correlations with pH. Previous studies have demonstrated that these microbial taxa can mediate Sb/As transformation processes in various environmental systems [19,44,45,46]. For example, *Luteimonas* has been reported to promote Sb mobilization in Sb-contaminated shooting range soils [45], while *Arthrobacter* can tolerate As toxicity and facilitate As removal from contaminated water [46]. These findings suggest that, during microorganism–soil interactions, these microbial groups may play important roles in Sb and As release and oxidation processes. In general, the above results suggest that *Arenimonas*, *Luteimonas*, *Arthrobacter*, and *Brevundimonas* are likely the key functional microorganisms responsible for Sb(III) and As(III) oxidation in soil, whereas *Devosia* and *Aminobacter* may contribute to Sb and As release by promoting the increase in soil pH.

## 4. Conclusions

Mining and smelting activities generate large amounts of tailings and residues, resulting in excessive Sb and As accumulation in the environment and posing severe risks to human health and ecosystem safety. Microorganisms play critical roles in regulating the environmental fate of Sb and As by mediating their migration and transformation processes. This study elucidated the biogeochemical pathways regulating Sb/As speciation and stability in the soil environment of antimony smelting areas. The results demonstrated that microorganisms accelerated the biogeochemical cycling of Sb and As by promoting their dissolution and release from antimony smelting soils through pH elevation, followed by the complete oxidation of released Sb(III) and As(III). A fraction of the dissolved Sb(V) and As(V) was re-immobilized through precipitation processes. Moreover, the dissolution and release of Sb and As facilitated the selective enrichment of microorganisms associated with Sb/As metabolism. These findings elucidate the interactions between Sb-As co-contaminated soils and microorganisms and highlight the important role of Sb/As-oxidizing microorganisms in controlling the migration and distribution of Sb and As, as they can reduce both the toxicity and mobility of Sb/As in soil solid phases. Given the ecological risks posed by the microbially enhanced release of Sb and As, standardized ecotoxicity testing using representative model organisms should be regarded as a critical priority for follow-up research.

## Figures and Tables

**Table 1:** Physicochemical characterization of the soil sample.

pH	Moisture (%)	Clay (%)	EC (μs cm^−1^)	TOC (%)	TN (g kg^−1^)	TP (g kg^−1^)	Fe (g kg^−1^)	Ca (g kg^−1^)	Na (g kg^−1^)	K (g kg^−1^)
7.39 ± 0.02	41.10 ± 1.30	7.92 ± 0.59	565.00 ± 19.29	10.48 ± 0.10	7.08 ± 0.35	8.42 ± 0.51	48.23 ± 3.36	81.79 ± 5.81	1.55 ± 0.19	4.38 ± 0.07

Data are presented as mean ± standard deviation (*n* = 3).

## Data Availability

The original contributions presented in this study are included in the article/Supplementary Materials. Further inquiries can be directed to the corresponding author(s).

## Supplementary Materials

The following supporting information can be downloaded at: https://www.mdpi.com/article/10.3390/toxics14090759/s1, Table S1: CDM composition used in this study; Table S2: The initial experimental conditions; Table S3: Sequential extraction scheme used in this study.
