# Supplementary material for: The Release and Transformation of Sb and As Induced by Microorganisms in Antimony Smelting Soil
Source: Toxics. 2026 Aug 26;14(9):759. doi: 10.3390/toxics14090759 (PMC13611151; doi:10.3390/toxics14090759)
Supplement: Supplementary file 1 [file toxics-14-00759-s001.zip › toxics-4504207-supplementary.pdf]

The release and transformation of Sb and As induced by microorganisms in antimony smelting soils

Yan Zheng [1,2], Zhihao Wu [2,3], Junhuan Wang [2,4,*], and Hong Hou [2,*]

[1] State Key Laboratory of Pollution Control and Resources Reuse, College of Environmental Science and Engineering, Tongji University, Shanghai 200092, China
[2] State Key Laboratory of Environmental Criteria and Risk Assessment, Chinese Research Academy of Environmental Sciences, Beijing 100012, China
[3] College of Water Sciences, Beijing Normal University, Beijing 100875, China
[4] School of Ocean Food and Biological Engineering, Jiangsu Ocean University, Lianyungang, Jiangsu, China
* Correspondence: 2025000084@jou.edu.cn and houhong@craes.org.cn

Supplementary Materials:

Table Captions

Table S1 CDM composition used in this study

Table S2 The initial experimental conditions

Table S3 Sequential extraction scheme used in this study

Table S1 CDM composition used in this study, which was based on Weeger et al. (1999)[1]

Formula	Concentration/ g L^{-1}
$MgSO_4 \cdot 7H_2O$	2.001
NH_4Cl	1.00
Na_2SO_4	0.994
$K_2HPO_4 \cdot 3H_2O$	0.013
$CaCl \cdot 2H_2O$	0.051
Na lactate（60 % w/w）	8.33

Note: [1] Weeger et al. (1999) is in the reference list in the main text.

Table S2 The initial experimental conditions

Step	pH	CDM (mL)	Soil (g)	Cell density (CFU mL^{-1})	Inoculum volume(mL)	Temperature (℃)	Aeration status
Inoculated microcosm	7.42	300	30	1.98×10^{7}	3	30	Aerobic
Abiotic control	7.42	300	30	0	0	30	Aerobic

Table S3 Sequential extraction scheme used in this study, which was based on Wenzel et al. (2001)[1]

Step	Target phase	Extractant	Extraction conditions	[g]:[mL]
F1	Easily exchangeable	0.05 M $(NH_4)_2SO_4$	4 h shaking, 20°C	1:25
F2	Specifically surface-bound	0.05 M $(NH_4)H_2PO_4$	16 h shaking, 20°C	1:25
F3	Amorphous hydrous oxide-bound	0.2 M NH_4-oxalate buffer, pH 3.25	4 h shaking 20°C in the dark	1:25
F4	Crystalline hydrous oxide-bound	0.2 M NH_4-oxalate buffer + 0.1 M ascorbic acid, pH 3.25	30 min, water basin at 96 ± 3°C daylight	1:25
F5	Residue	HCl-HF-H_2O_2-HNO_3	-	-

Note: [1] Wenzel et al. (2001) is in the reference list in the main text.
